# 4‐HIAA Blocks Methamphetamine‐Induced Conditioned Place Preference in Mice Through Modulation of the 5‐HT Pathway in the Nucleus Accumbens

**DOI:** 10.1111/adb.70063

**Published:** 2025-07-01

**Authors:** Yanan Wu, Ju Ran, Jinqiu Mo, Jing Wang

**Affiliations:** ^1^ Tarim University Alaer Xinjiang China; ^2^ Ruijin Hospital, Shanghai Jiaotong University School of Medicine Shanghai China; ^3^ Center for Excellence in Brain Science and Intelligence Technology Shanghai China

**Keywords:** 4‐hydroxyindole‐3‐acetic acid, conditioned place preference, methamphetamine, nucleus accumben, serotonin

## Abstract

4‐hydroxyindole‐3‐acetic acid (4‐HIAA) is a metabolite of psilocin. Here, we explored the ability of 4‐HIAA to cross the blood–brain barrier and its potential effects on methamphetamine (METH)‐induced conditioned place preference (CPP) in mice. Treatment with 1‐mg/kg 4‐HIAA inhibited CPP formation during the acquisition phase, promoted METH extinction and inhibited METH relapse. Furthermore, the regulatory effect of 4‐HIAA on METH was underscored by altered 5‐HT expression in the nucleus accumbens. Collectively, our findings provide novel insights into the molecular mechanisms of the 4‐HIAA‐induced blockade of the acquisition, extinction and reinstatement of METH‐induced CPP.

## Graphical abstract

4‐HIAA crosses the blood–brain barrier. Treatment (1‐mg/kg) inhibited CPP acquisition, promoted extinction and prevented relapse. These behavioural effects were associated with changes in nucleus accumbens 5‐HT expression.

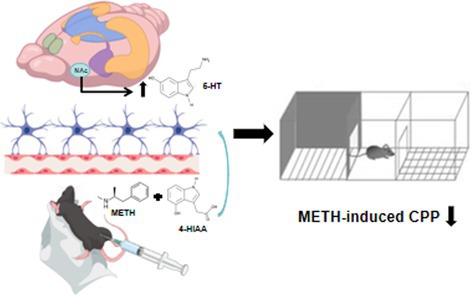

**Abbreviations**
4‐HIAA: 4‐hydroxyindole‐3‐acetic acid
5‐HIAA: 5‐hydroxyindole‐3‐acetic acid
5‐HT: serotonin
ADH: alcohol dehydrogenase
ALDH: aldehyde dehydrogenase
CPP: conditioned place preference
CSF: cerebrospinal fluid
ELISA: enzyme‐linked immunosorbent assay
METH: methamphetamine
NAc: nucleus accumbens
PFC: prefrontal cortex
VTA: ventral tegmental area

## 1 Introduction

Methamphetamine (METH) has neurotoxic properties [1], and its extended use can induce damage to serotonergic neurons, resulting in significant brain damage and manifestations of various neuropsychological disorders, including depression, anxiety and cognitive impairment [2]. The intraperitoneal administration of 10‐mg/kg METH results in a notable reduction in the concentrations of cortical serotonin (5‐HT) after 3 h, with the effect persisting for up to 24 h, indicating its potential for long‐lasting reduction in indoleamine concentrations [3]. Similarly, during METH withdrawal in animal studies, the synaptic monoamine levels diminish in the limbic brain nuclei and the administration of non‐contingent doses of METH during this period leads to a decrease in neurotransmitter release compared to drug‐naïve animals.

Notably, the regions affected by METH‐induced serotonergic damage encompass, but are not restricted to, the perirhinal cortex, hippocampus, anterior cingulate cortex, caudate nucleus, nucleus accumbens (NAc) and septum [4]. Additionally, repeated METH administration significantly decreases the concentration of 5‐HT and its metabolite 5‐hydroxyindole‐3‐acetic acid (5‐HIAA) in the caudate area [5]. 5‐HIAA is the principal metabolite of 5‐HT and is predominantly present in the cerebrospinal fluid, serving as an indicator of the amount of 5‐HT that has undergone metabolism and clearance from the synaptic cleft [6]. Alterations in 5‐HIAA levels have been linked to various psychiatric disorders, including depression, anxiety and impulsivity [7, 8]. Therefore, targeting the 5‐HT metabolic pathway is a potentially viable therapeutic approach against METH addiction. A recent study indicated that fluoxetine, a selective serotonin reuptake inhibitor, can inhibit METH‐induced locomotor sensitisation [9].

4‐Hydroxyindole‐3‐acetic acid (4‐HIAA), a primary metabolite of psilocin [10], diverges pharmacologically from its parent compound. Psilocin mediates its psychedelic effects via direct agonism of 5‐HT receptors, with concomitant increases in extracellular 5‐HT concentrations within mesocorticolimbic pathways [11]. In contrast, 4‐HIAA exhibits negligible binding affinity for canonical 5‐HT receptor subtypes [12], and its functional roles in the central nervous system (CNS) remain poorly characterised. Limited evidence suggests that 4‐HIAA serves as a structural moiety in argiotoxins, neuroactive spider venom components that induce neuromuscular paralysis at submicromolar concentrations [13]. 4‐HIAA contains an indole moiety, and existing literature reports that certain indole‐containing compounds can modulate serotonergic signalling pathways [14, 15]. This structural analogy raises speculative questions about whether 4‐HIAA may influence 5‐HT metabolism or related processes indirectly, though empirical validation of such mechanisms remains absent in the current literature.

Based on the above research findings, we hypothesised that 4‐HIAA may have an impact on METH‐induced behavioural alterations through its interaction with 5‐HT. Therefore, this study aimed to investigate the ability of 4‐HIAA to traverse the blood–brain barrier and examine its influence on the expression of 5‐HT in the prefrontal cortex (PFC), NAc and ventral tegmental area (VTA). Furthermore, the study aimed to elucidate the molecular mechanisms underlying the behavioural effects of 4‐HIAA on METH‐induced changes in mice.

## 2 Materials and Methods

### 2.1 Animals

All experiments were conducted in strict accordance with ARRIVE guidelines. Eight‐week‐old male C57BL/6J mice were procured from Chengdu Dossy Experimental Animal Company. The housing conditions maintained a temperature range of 20°C–24°C, humidity levels between 40% and 60% and followed a 12/12‐h light/dark cycle; these conditions were precisely documented to ensure animal well‐being and reproducibility of the experimental environment. To minimise the influence of environmental factors on protein expression, a week‐long acclimation period was provided to all animals prior to the commencement of the experiments. All mice were euthanised by CO_2_ asphyxiation, followed by cervical dislocation (AVMA euthanasia guidelines). This study was approved by the Institutional Animal Care and Use Committee of Tarim University. All animal procedures adhered to the internationally accepted standards for animal research following the 3Rs principle.

### 2.2 Drugs

METH powder was purchased from China Pharmaceutical and Biological Products (PR China) and 4‐HIAA was purchased from Cayman Chemical Company (USA). In the METH‐induced conditioned place preference (CPP) model, a 1‐mg/kg dose was used for both METH and 4‐HIAA. All the drugs were administered via intraperitoneal injection. A previous study indicated that 1‐mg/kg psilocin suppressed METH‐induced acquisition of CPP [16]. Because a large proportion of psilocin is metabolised to 4‐HIAA by monoamine oxidase and aldehyde dehydrogenase, 1‐mg/kg 4‐HIAA was chosen for this study [12, 17].

### 2.3 High‐Performance Liquid Chromatography (HPLC) Conditions

An LC‐2030C 3D Plus instrument (Shimadzu, Kyoto, Japan) was used for HPLC. A C18 column was used for chromatographic separation. Water (Solvent A) and methanol (Solvent B) were used as mobile phases for linear gradient elution. Each sample was tested for 20 min. The concentration of Solvent B was maintained at 5% for the first 4.00 min, and then increased to 90% from 4.00 to 10.00 min, held at 90% from 10.00 to 15.00 min, decreased to 5% from 15.00 to 15.50 min and held at 5% from 15.50 to 20.00 min for re‐equilibration. The flow rate was kept constant at 0.20 mL/min, and the column temperature was set at 30°C. After ultrasound, the sample was precipitated with acetonitrile and filtered using a 0.22‐μm nylon membrane. In this experiment, four groups were established: the first group was a 0.1‐mg/mL 4‐HIAA standard solution; the second, third and fourth groups were whole mouse brain tissue harvested 30 min after intraperitoneal injection of saline, saline along with supplementation of 5 μL of 0.1‐mg/mL 4‐HIAA standard solution, and 1‐mg/kg 4‐HIAA, respectively. Each sample injection volume was 20 μL.

### 2.4 CPP Model

On the first day of the experiment, a pre‐test lasting 15 min was conducted on the mice. Mice exhibiting abnormal activity characterised by low motor activity (fewer than 20 shuttles) or spending more than 600 s on either side were excluded from further analysis.

Modelling was performed for each phase of the study. During the CPP acquisition phase (Days 2–9), specific injection and compartment placement protocols were followed. On Days 2, 4, 6 and 8, all groups received saline injections and were placed in a black compartment for 45 min. On Days 3, 5, 7 and 9, different treatments were administered based on the group: The saline group received saline injections; the drug group received 1‐mg/kg 4‐HIAA injections; the METH group received METH injections; and the METH with drug intervention group received 1‐mg/kg 4‐HIAA injections 30 min prior to METH administration (based on a recent study that reported 4‐HIAA *t*_max_ = 0.30 ± 0.11 h in mice [12], and another study, wherein treatment of Alzheimer's disease model mice with 5‐HIAA, a chemical analogue of 4‐HIAA, elicited remarkable differences 30 min after the drug administration [18]). All groups were then placed in the white compartment for 45 min. On post‐test day (Day 10), all mice were allowed to freely explore both compartments for 15 min (Figure 2A). Following the completion of the CPP acquisition phase, tissue samples from the PFC, NAc and VTA were collected and subsequently stored at −80°C for further analysis.

Modelling was performed for each phase of the study. The extinction phase (Days 11–22) involved four groups: saline, 4‐HIAA, METH‐Ext1 and METH‐Ext2. The METH group with the acquisition phase was divided into METH‐Ext1 and METH‐Ext2 groups. On Day 11, all four groups received saline injections and were placed in a black compartment for 45 min. On Day 12, the saline and METH‐Ext1 groups received saline injections, whereas the 4‐HIAA and METH‐Ext2 groups received 4‐HIAA injections. All groups were placed in the white compartment for 45 min. On Day 13, all the mice were allowed to freely explore both compartments for 15 min. This protocol was repeated thrice from Days 14 to 22 (Figure 3A).

Modelling was performed for each phase of the study. Six groups were tested during the reinstatement phase: saline, 4‐HIAA, METH‐Rein1, METH‐Rein2, METH‐Rein3 and METH‐Rein4. The METH‐Ext1 group was further divided into METH‐Rein1 and METH‐Rein2 groups. The METH‐Rein1 group received METH injections, whereas the METH‐Rein2 group received 1‐mg/kg 4‐HIAA injections prior to METH administration. The METH‐Ext2 group was further divided into METH‐Rein3 and METH‐Rein4 groups. The METH‐Rein3 group received METH injections, and the METH‐Rein4 group received 1‐mg/kg 4‐HIAA injections prior to METH administration. Subsequently, the mice were allowed to freely explore both compartments for 15 min (Figure 4A). Following the completion of the CPP reinstatement phase, tissue samples from the PFC, NAc and VTA were collected and subsequently stored at −80°C for further analysis.

CPP scores were calculated as the time spent in the METH‐paired compartment, minus the time spent in the saline‐paired compartment.

### 2.5 Enzyme‐Linked Immunosorbent Assay (ELISA)

ELISA was used to detect the levels of 5‐HT in the PFC, NAc and VTA dissected from the METH‐induced CPP acquisition or reinstatement groups. The ELISA kits were purchased from Elabscience Biotechnology (Wuhan, China). Briefly, 50 μL of the sample and 50 μL of the prepared biotinylated antibody working solution were added into each well of the microplate, which was then sealed with a film and incubated at 37°C for 45 min to facilitate binding reactions. Thereafter, the microplate was flicked to empty the wells and dried on lint, followed by addition of 350 μL of washing buffer to each well, incubation for 1 min, aspiration of the liquid and pat drying. This cycle was performed thrice to remove unbound substances. Thereafter, 100 μL of HRP‐enzyme conjugate working solution was added to each well and the microplate was resealed and incubated at 37°C for 30 min. The wells were then emptied and washed five times following the same soaking, aspirating and drying steps as before to reduce background. Subsequently, 90 μL of TMB substrate solution was added to each well, followed by covering of the microplate with a light‐blocking film and incubation at 37°C in the dark for approximately 15 min for colour development. Post incubation, 50 μL of stop solution was added to halt the reaction, and the optical density (OD) at 450 nm was immediately measured by Multiskan FC Microplate Reader (Thermo Scientific, USA) and the data were recorded.

### 2.6 Statistical Analysis

SPSS 22.0 (SPSS, USA) was used for all statistical analyses. Acquisition and extinction of METH‐induced CPP data were analysed using a two‐way ANOVA. Reinstatement of METH‐induced CPP and ELISA data were analysed using one‐way ANOVA. All post hoc pairwise comparisons were performed using Bonferroni's test. *p* values < 0.05 were considered statistically significant.

## 3 Results

### 3.1 Ability of 4‐HIAA to Cross the Blood–Brain Barrier

**FIGURE 1:**
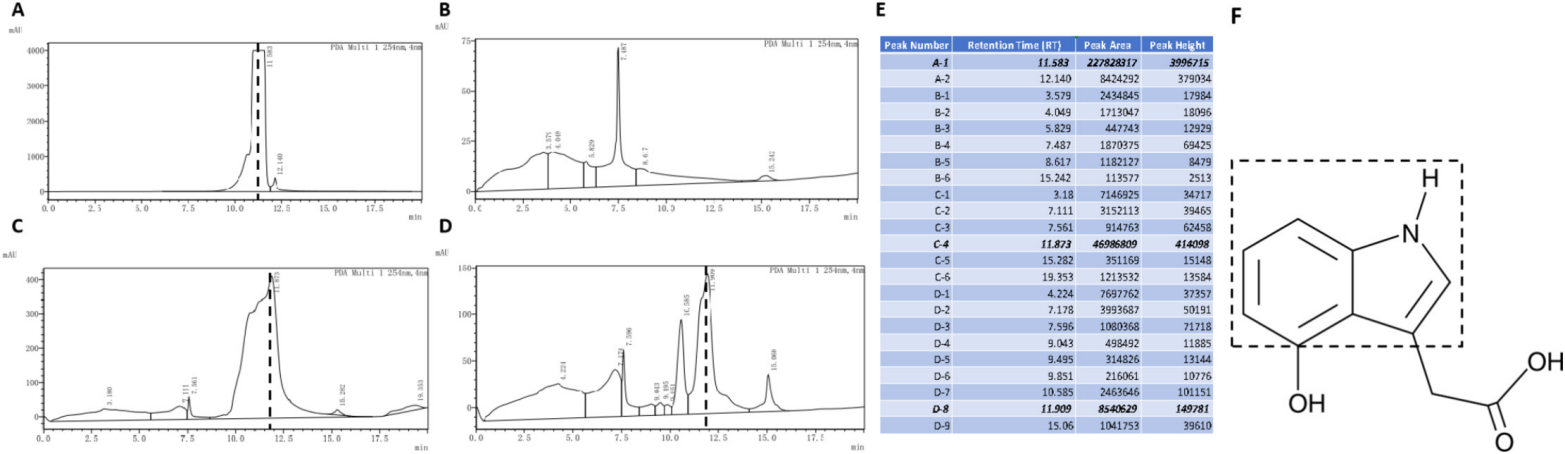
The ability of 4‐HIAA through the blood–brain barrier in mice. (A) The result of 4‐HIAA standard group in HPLC. (B) The result of saline brain group in HPLC. (C) The result of saline brain with 4‐HIAA standard group in HPLC. (D) The result of 4‐HIAA intraperitoneal injection group in HPLC. (E) The detail of all peaks in four groups. (F) The structural diagram of 4‐HIAA, with its indole moiety enclosed within a dashed‐line box.

The retention time of 4‐HIAA was initially established by analysing the standard sample, as illustrated in Figure 1A, using the specified detection method. The presence of 4‐HIAA in other samples was confirmed by identifying peaks at the same retention time, with a margin of error not exceeding 2%. This study was conducted as a qualitative investigation, with samples collected 30 min following intraperitoneal administration of either 4‐HIAA (1 mg/kg) or saline (*n* = 3). Brain tissues were subsequently harvested for HPLC analysis. The retention time of 4‐HIAA was determined to be 11.75 ± 0.17 min, as evidenced by the chromatograms of the 4‐HIAA standard (Figure 1A), the saline brain sample spiked with 4‐HIAA standard (Figure 1C) and the brain sample from mice administered 4‐HIAA via intraperitoneal injection (Figure 1D). Notably, 4‐HIAA was not detected in the saline‐only brain group (Figure 1B). Figure 1E presents the chromatographic profiles of all experimental groups, with 4‐HIAA identified as the primary compound of interest. Unassigned peaks observed in the chromatograms are likely attributable to matrix‐derived interferences.

### 3.2 Regulatory Effects of 4‐HIAA on Acquisition of METH‐Induced CPP

**FIGURE 2:**
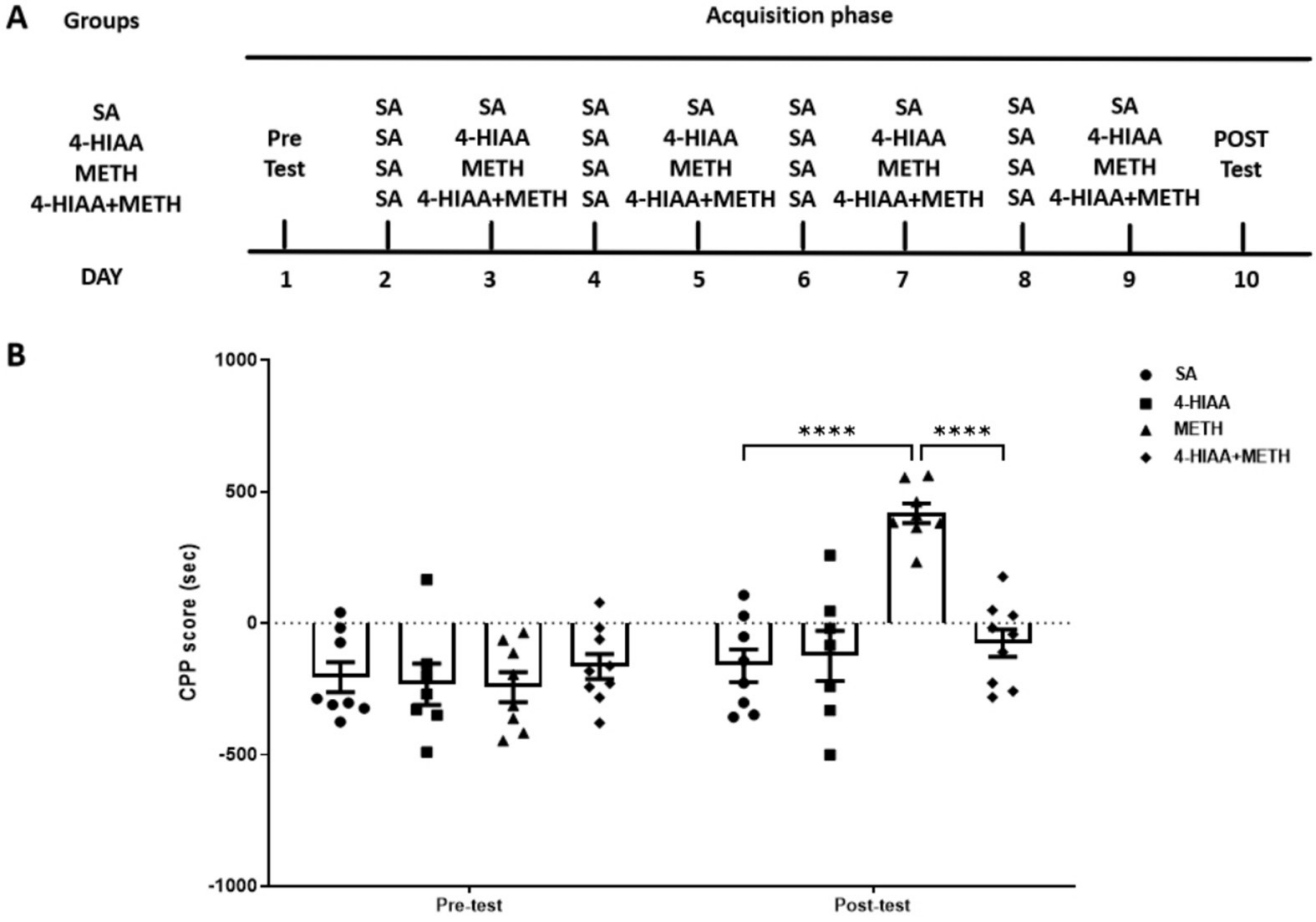
Effects of 1‐mg/kg 4‐HIAA on the acquisition of METH‐induced CPP. 4‐HIAA inhibited CPP acquisition. Data are presented as mean ± SEM (*n* = 7–9). ****p < 0.0001.

A dose of 1‐mg/kg 4‐HIAA was injected into the METH‐induced CPP model (*n* = 7–9). We observed significant interaction (*F*_(3,28)_ = 11.08, *p* < 0.0001) and main effects of time (*F*_(1,28)_ = 26.11, *p* < 0.0001) and treatment (*F*_(3,28)_ = 8.939, *p* < 0.001) on the acquisition phase. The post‐test results indicated that the CPP scores of the METH group were significantly higher than those of the saline group (*p* < 0.0001). Furthermore, the injection of 1‐mg/kg 4‐HIAA before METH administration significantly inhibited the increase in CPP scores in the METH group (*p* < 0.0001), and there was no difference compared to that of the saline group. No significant difference in CPP scores was observed between the 1‐mg/kg 4‐HIAA and saline groups, *n* = 7–9 (Figure 2B), indicating that 4‐HIAA alone is incapable of inducing either CPP or conditioned place aversion (CPA).

### 3.3 Regulatory Effects of 4‐HIAA on Extinction of METH‐Induced CPP

**FIGURE 3:**
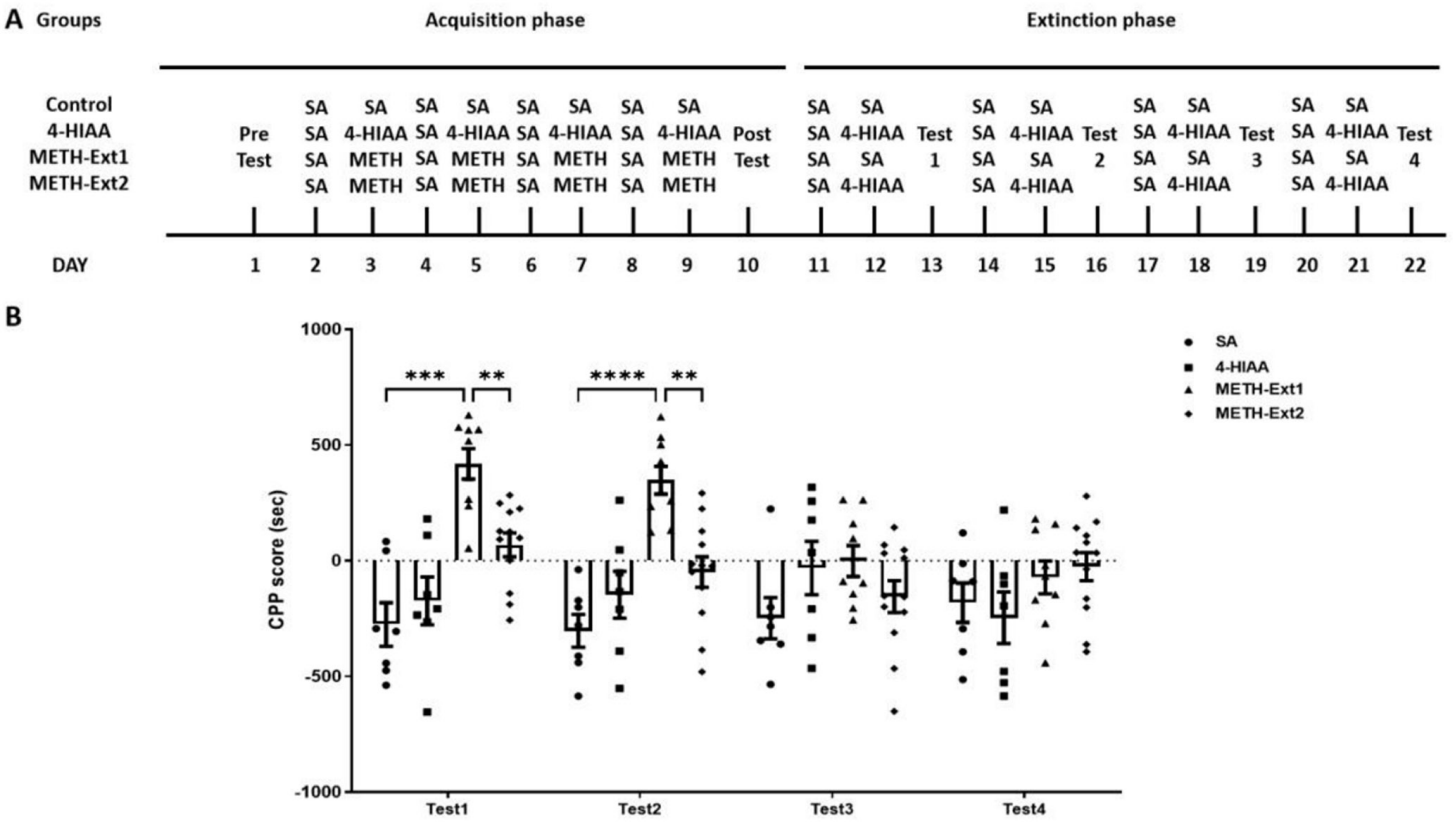
Effects of 1‐mg/kg 4‐HIAA on the extinction of METH‐induced CPP. 4‐HIAA on the extinction of CPP. Data are presented as mean ± SEM (*n* = 7–12). **p < 0.01, ***p < 0.001, ****p < 0.0001.

In the extinction phase, the METH group was divided into two groups: The METH‐Ext1 group was injected with saline, and METH‐Ext2 group was injected with 1‐mg/kg 4‐HIAA (*n* = 7–12). The saline and 4‐HIAA groups underwent continuous training. We observed significant interaction (*F*_(9,93)_ = 5.158, *p* < 0.0001) and main effects of time (*F*_(2.512, 77.86)_ = 4.152, *p* < 0.05) and treatment (*F*_(3,31)_ = 10.04, *p* < 0.0001) on the extinction phase. No significant differences in CPP scores were observed between the 4‐HIAA and control groups for each test. The METH‐Ext1 group exhibited significant differences in Test 1 (*p* < 0.001) and Test 2 (*p* < 0.0001) compared with the saline group. The METH‐Ext1 group showed CPP regression after three extinction training sessions. The 4‐HIAA accelerated the extinction of METH. The METH‐Ext2 groups exhibited significant differences in Test 1 (*p* < 0.01) and Test 2 (*p* < 0.01) compared to the METH‐Ext1 group and no significant difference compared to the saline group, *n* = 7–12 (Figure 3B).

### 3.4 Regulatory Effects of 4‐HIAA on Reinstatement of METH‐Induced CPP

**FIGURE 4:**
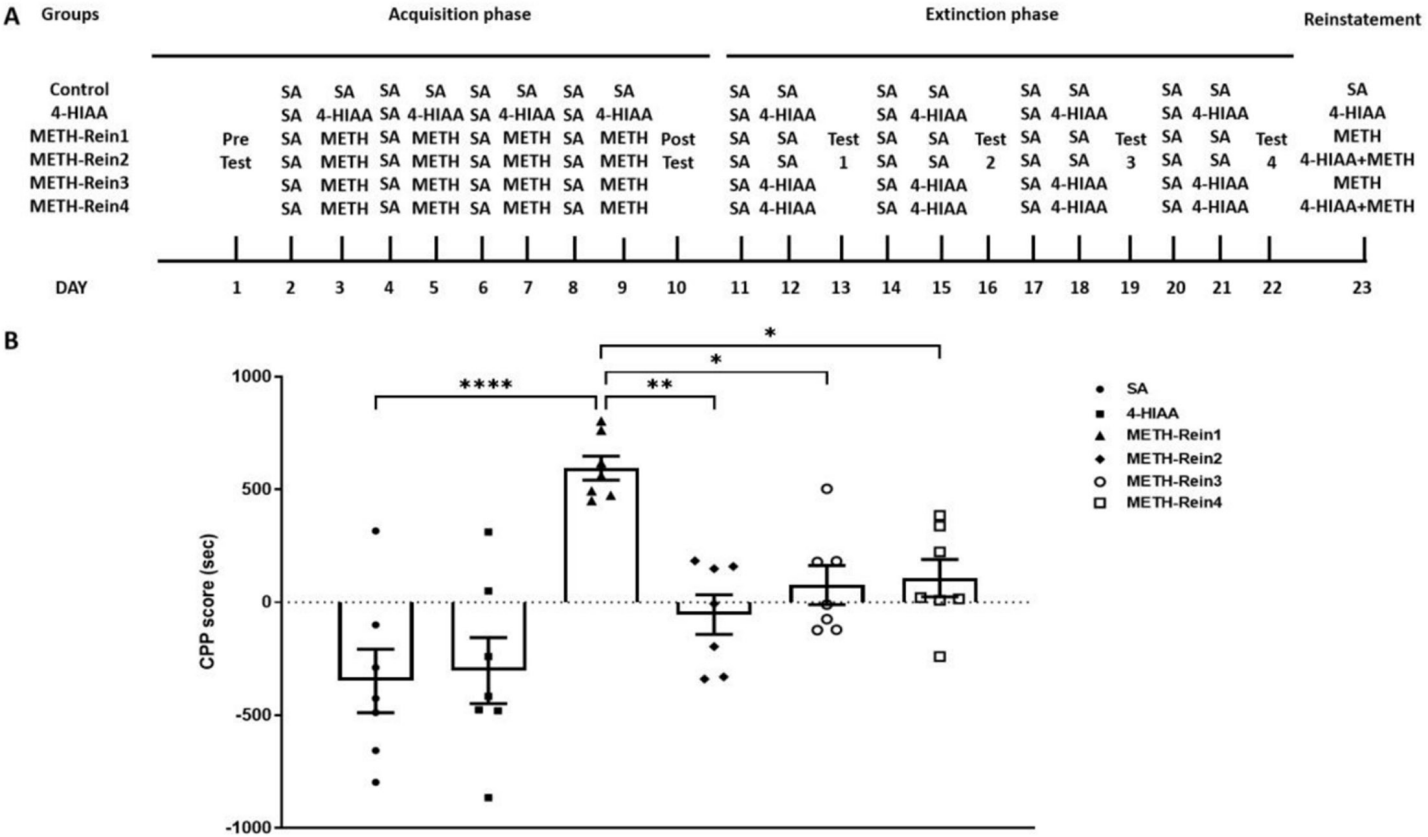
Effects of 1‐mg/kg 4‐HIAA on the reinstatement of METH‐induced CPP. 4‐HIAA on the reinstatement of CPP. Data are presented as mean ± SEM (*n* = 7). *p < 0.05, **p < 0.01, ****p < 0.0001.

In the reinstatement phase, the METH‐Ext1 group was further divided into the METH‐Rein1 and METH‐Rein2 groups, and the METH‐Ext2 group was further divided into the METH‐Rein3 and METH‐Rein4 groups (*n* = 7). Mice in the METH‐Rein1 and METH‐Rein3 groups were injected with METH, whereas those in the METH‐Rein2 and METH‐Rein4 groups were injected with 1‐mg/kg 4‐HIAA 30 min before METH injection. We observed a significant difference in the CPP reinstatement phase (*F*_(5,36)_ = 10.65, *p* < 0.0001). The CPP scores of the METH‐Rein1 group were significantly higher than those of the saline group (*p* < 0.0001). 4‐HIAA acute and chronic administration could inhibit the reinstatement of METH‐induced CPP, in which the CPP scores of the METH‐Rein2, METH‐Rein3 and METH‐Rein4 groups were significantly lower than those of the METH‐Rein1 group (*p* < 0.01, *p* < 0.05, *p* < 0.05, respectively) and were not significantly different from those of the saline group, *n* = 7 (Figure 4B).

### 3.5 5‐HT Expression in the PFC, NAc and VTA in METH‐Induced CPP Acquisition

**FIGURE 5:**
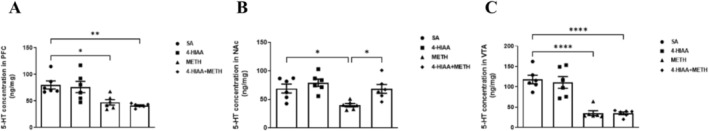
ELISA of 5‐HT in the PFC, NAc and VTA after the acquisition of METH‐induced CPP. Data are presented as the mean ± SEM (*n* = 6). *p < 0.05, **p < 0.01, ****p < 0.0001.

Expression levels of 5‐HT in the PFC, NAc and VTA were measured (*n* = 6). Significant differences were observed in the PFC (*F*_(3,20)_ = 7.955, *p* < 0.01), NAc (*F*_(3,20)_ = 6.699, *p* < 0.01) and VTA (*F*_(3,20)_ = 23.07, *p* < 0.0001). The post‐test indicated that 5‐HT in the METH group was significantly decreased compared to that in the saline group in the PFC (*p* < 0.05), NAc (*p* < 0.05) and VTA (*p* < 0.0001). Furthermore, the injection of 1‐mg/kg 4‐HIAA before METH significantly increased the decrease in 5‐HT levels in the NAc compared to those in the METH group (*p* < 0.05); however, no significant difference was observed in the PFC and VTA, *n* = 6 (Figure 5).

### 3.6 5‐HT Expression in the PFC, NAc and VTA in METH‐Induced CPP Reinstatement

**FIGURE 6:**
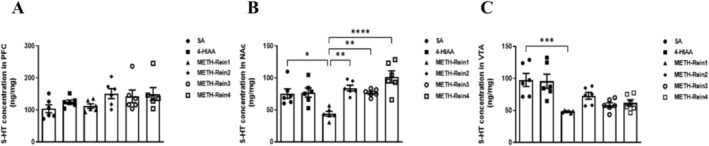
ELISA of 5‐HT in the PFC, NAc and VTA after the reinstatement of METH‐induced CPP. Data are presented as the mean ± SEM (*n* = 6). *p < 0.05, **p < 0.01, ***p < 0.001, ****p < 0.0001.

Expression levels of 5‐HT in the PFC, NAc and VTA were measured (*n* = 6). We observed significant differences in the NAc (*F*_(5,30)_ = 9.063, *p* < 0.0001) and VTA (*F*_(5,30)_ = 8.508, *p* < 0.0001), but no significant difference in the PFC. Post‐test indicated that 5‐HT levels in the METH‐Rein1 group were significantly decreased compared to those in the saline group in the NAc (*p* < 0.05) and VTA (*p* < 0.001). Furthermore, both on extinction and reinstatement of METH‐induced CPP, the injection of 1 mg/kg 4‐HIAA before METH significantly mitigated the decrease in 5‐HT level in the NAc compared to that in the METH group (METH‐Rein2, *p* < 0.01; METH‐Rein2, *p* < 0.01; METH‐Rein3, *p* < 0.0001); however, no significant difference was observed between the VTA samples, *n* = 6 (Figure 6).

## 4 Discussion

The CPP is a widely utilised and trustworthy behavioural model employed in the study of reward effects induced by various drugs of abuse [19]. Prolonged exposure to addictive substances such as METH activates reward circuits spanning the VTA to the NAc and PFC [20]. In this study, regarding acquisition and reinstatement of METH‐induced CPP, the 5‐HT levels in the METH group were significantly lower than those in the saline group in the NAc and VTA. A recent study also indicated that, in both human and animal models, METH can induce neurotoxicity, which is evidenced by the reduction of serotonin axon terminal markers in the PFC, NAc and VTA [21, 22]. Additionally, chronic METH use can lead to long‐term deficits in serotonin transmission [23].

In this study, 4‐HIAA was able to traverse the blood–brain barrier. Similarly, recent studies have demonstrated that 5‐HIAA serves as a derivative of serotonin and that its passage through the blood–brain barrier has the potential to modulate the overall serotonin equilibrium in the brain [24]. Variations in 5‐HIAA concentration may mirror shifts in serotonin turnover and metabolism, thereby influencing the accessibility of serotonin for neurotransmission [25].

This study demonstrates that 4‐HIAA administered at a dose of 1 mg/kg inhibits METH‐induced CPP. Furthermore, it facilitates the extinction of METH‐associated behaviours and prevents METH relapse. Recent animal studies have revealed that tryptamine‐type psychedelics regulate CPP. For example, psilocin suppresses the METH‐induced acquisition of conditioned place preference via D2R‐mediated ERK signalling [16]. Additionally, DMT pretreatment successfully inhibits ethanol‐induced CPP [26]. Tryptamines are characterised by an indole structure consisting of a six‐membered benzene ring fused to a five‐membered pyrrole ring. Many bioactive compounds, including endogenous substances such as serotonin and melatonin as well as drugs such as indomethacin, ondansetron, tadalafil, frovatriptan and various tryptamines, incorporate an indole scaffold. Moreover, 4‐HIAA belongs to the indoleacetic acid category, which is a class of substances containing an indole scaffold. These compounds are closely related to the endogenous neurotransmitter, 5‐HT, which contains a hydroxyl group at the C5 position. Conventionally, 5‐HIAA and 4‐HIAA are regarded as biologically inert products. Nevertheless, emerging evidence challenges this view, suggesting that both 5‐HT and 5‐HIAA lead to a comparable reduction in RAS/MAPK signalling, with 5‐HIAA exerting a more potent effect than 5‐HT [27]. Similarly, in the present study, 4‐HIAA was shown to exert biological effects.

In the present study, we demonstrated that the regulatory effects of 4‐HIAA on METH were accompanied by changes in the expression of 5‐HT in the NAc. Consistent with previous research, it has been demonstrated that the direct infusion of 5‐HT inhibited METH‐induced locomotor activity, stereotypies, turning behaviour and extra‐pyramidal functions [28]. Structurally, 4‐HIAA shares homology with endogenous indole derivatives, such as indole‐3‐acetic acid [14] and indoleacetaldehydes [15], compounds that regulate serotonergic signalling pathways. In addition, our results indicate that the regulation of 5‐HT in the NAc by 4‐HIAA is remarkably significant. Further research demonstrated that repeated high‐dose exposure to METH causes 5‐HT depletion in the NAc [21]. Moreover, input‐specific effects on excitatory transmission to the NAc contribute to distinct behavioural modulation of 5‐HT release [29].

This study has some limitations. Although we conducted an acute administration experiment and observed that 4‐HIAA effectively inhibited METH‐induced hyperactivity (Figure S1), 4‐HIAA had no significant impact on cognitive function (Figure S2). It is important to note that METH elicits a broad spectrum of appetitive or aversive effects, which influence the risk of continued drug use and addiction [30]. In the current study, the potential adverse effects of 4‐HIAA on METH levels were not investigated. Additionally, it is noteworthy that 4‐HIAA alone did not induce CPP or CPA in this experimental paradigm.

Future research should focus on comprehensive pharmacokinetic and safety evaluations to systematically assess potential interactions between 4‐HIAA and METH metabolism, neurotoxicological risks and off‐target behavioural or physiological effects. Key areas of investigation should include determining the retention duration of 4‐HIAA in the brain and quantifying its recovered concentration in brain tissue. Additionally, rigorous characterisation of dose–response relationships is essential to establish optimal dosing regimens, define therapeutic windows and evaluate the long‐term sustainability of 4‐HIAA's anti‐addiction efficacy through well‐designed longitudinal studies.

## 5 Conclusion

In conclusion, our current study findings indicate that the administration of 1‐mg/kg 4‐HIAA can effectively inhibit CPP formation, promote METH extinction and prevent METH relapse. The regulatory effects of 4‐HIAA on METH are mediated, at least in part, by the altered expression of 5‐HT in the NAc. Therefore, 4‐HIAA may block METH‐induced CPP in mice by modulating 5‐HT signalling in the NAc, which may pave the way for the development of novel interventions targeting the serotonin pathway in substance abuse disorders.

## Author Contributions

**Jing Wang:** funding acquisition, methodology and Writing – review and editing. **Yanan Wu:** investigation, methodology and validation. **Jinqiu Mo:** funding acquisition, formal analysis and writing – original draft. **Ju Ran:** data curation and funding acquisition. All data were generated in‐house, and no paper mill was used. All authors agree to be accountable for all aspects of work, ensuring integrity and accuracy.

## Ethics Statement

All mice were fed and housed according to the Guidelines for the Care and Use of Laboratory Animals issued by the National Institutes of Health, USA. Ethical approval for this research was obtained from the Institutional Animal Care and Use Committee of Tarim University.

## Conflicts of Interest

The authors declare no conflicts of interest.

## Supporting information


**Figure S1** **Effects of 1 mg/kg 4‐HIAA on the acute METH administration model.** Data are presented as the mean ± SEM (*n* = 7–8). *p < 0.05, ****p < 0.0001. F _(3,25)_ = 41.86, *p* < 0.0001. The results demonstrated that the METH group exhibited a significant increase in activity compared to the saline group (*p* < 0.0001). Furthermore, administration of 1 mg/kg 4‐HIAA significantly attenuated METH‐induced hyperactivity in mice, with the difference being statistically significant compared to the METH group *(p* < 0.05).
**Figure S2. Effects of acute 1 mg/kg 4‐HIAA on novel object recognition**. According to the experimental procedure of CPP, the interval between each drug administration is 24 h. We detected the novel object recognition 24 h after the training session. Data are presented as the mean ± SEM (*n* = 8). The results indicated no significant difference between the saline group and the 4‐HIAA group.

## Data Availability

The datasets used and analysed during the current study are available from the corresponding author on reasonable request.
