# Supplementary material for: 4‐HIAA Blocks Methamphetamine‐Induced Conditioned Place Preference in Mice Through Modulation of the 5‐HT Pathway in the Nucleus Accumbens
Source: Addict Biol. 2025 Jul 1;30(7):e70063. doi: 10.1111/adb.70063 (PMC12210140; doi:10.1111/adb.70063)
Supplement: Supplementary file 1 — Figure S1 Effects of 1 mg/kg 4‐HIAA on the acute METH administration model. Data are presented as the mean ± SEM (n = 7–8). *p < 0.05, ****p < 0.0001. F (3,25) = 41.86, p < 0.0001. The results demonstrated that the METH group exhibited a significant increase in activity compared to the saline group (p < 0.0001). Furthermore, administration of 1 mg/kg 4‐HIAA significantly attenuated METH‐induced hyperactivity in mice, with the difference being statistically significant compared to the METH group (p < 0.05). Figure S2. Effects of acute 1 mg/kg 4‐HIAA on novel object recognition. According to the experimental procedure of CPP, the interval between each drug administration is 24 h. We detected the novel object recognition 24 h after the training session. Data are presented as the mean ± SEM (n = 8). The results indicated no significant difference between the saline group and the 4‐HIAA group. [file ADB-30-e70063-s001.doc]

**4-HIAA Blocks Methamphetamine-Induced Conditioned Place Preference in Mice through Modulation of the 5-HT Pathway in the Nucleus Accumbens**

Yanan Wu ^1, #^, Ju Ran ^1, #^, Jinqiu Mo ^1, *^, Jing Wang ^2, 3, *^

1 Tarim University, Alaer, Xinjiang, China

2 Ruijin Hospital, Shanghai Jiaotong University School of Medicine, Shanghai Jiaotong, Shanghai, China

3 Center for Excellence in Brain Science and Intelligence Technology, Shanghai, China

*Corresponding authors at: E-mail addresses: [18160032290@163.com](mailto:18160032290@163.com) (Jinqiu Mo), [2858137410@qq.com](mailto:2858137410@qq.com) (Jing Wang).

^#^Contributed equally.


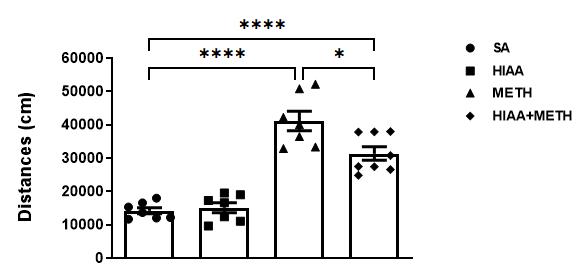


**Figure S1.** **Effects of 1 mg/kg 4-HIAA on the acute METH administration model.** Data are presented as the mean ± SEM (n =7-8). **P* < 0.05, *****P* < 0.0001. F _(3,25)_ = 41.86, *P* < 0.0001. The results demonstrated that the METH group exhibited a significant increase in activity compared to the saline group (*P* < 0.0001). Furthermore, administration of 1 mg/kg 4-HIAA significantly attenuated METH-induced hyperactivity in mice, with the difference being statistically significant compared to the METH group *(P* < 0.05).


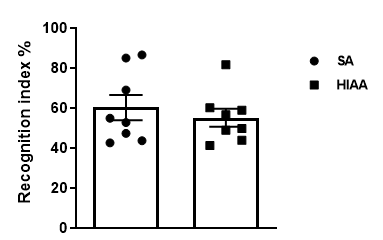


**Figure S2.** **Effects of acute 1 mg/kg 4-HIAA on novel object recognition.** According to the experimental procedure of CPP, the interval between each drug administration is 24 hours. We detected the novel object recognition 24 hours after the training session.Data are presented as the mean ± SEM (n =8). The results indicated no significant difference between the saline group and the 4-HIAA group.
